# Sheldon spectrum and the plankton paradox: two sides of the same coin—a trait-based plankton size-spectrum model

**DOI:** 10.1007/s00285-017-1132-7

**Published:** 2017-05-25

**Authors:** José A. Cuesta, Gustav W. Delius, Richard Law

**Affiliations:** 1 Grupo Interdisciplinar de Sistemas Complejos (GISC), UC3M-BS Institute of Financial Big Data (IFiBiD), Departamento de Matemáticas, Universidad Carlos III de Madrid, Madrid, Spain; 2 Instituto de Biocomputación y Física de Sistemas Complejos (BIFI), Universidad de Zaragoza, Zaragoza, Spain; 3 Department of Mathematics and the York Centre for Complex Systems Analysis, University of York, York, UK

**Keywords:** Plankton, Coexistence, Allometry, Size-spectrum, Scale-invariance, Cell division, 92D40, 92D25, 92C37

## Abstract

The Sheldon spectrum describes a remarkable regularity in aquatic ecosystems: the biomass density as a function of logarithmic body mass is approximately constant over many orders of magnitude. While size-spectrum models have explained this phenomenon for assemblages of multicellular organisms, this paper introduces a species-resolved size-spectrum model to explain the phenomenon in unicellular plankton. A Sheldon spectrum spanning the cell-size range of unicellular plankton necessarily consists of a large number of coexisting species covering a wide range of characteristic sizes. The coexistence of many phytoplankton species feeding on a small number of resources is known as the Paradox of the Plankton. Our model resolves the paradox by showing that coexistence is facilitated by the allometric scaling of four physiological rates. Two of the allometries have empirical support, the remaining two emerge from predator–prey interactions exactly when the abundances follow a Sheldon spectrum. Our plankton model is a scale-invariant trait-based size-spectrum model: it describes the abundance of phyto- and zooplankton cells as a function of both size and species trait (the maximal size before cell division). It incorporates growth due to resource consumption and predation on smaller cells, death due to predation, and a flexible cell division process. We give analytic solutions at steady state for both the within-species size distributions and the relative abundances across species.

## Introduction

Gaining a better understanding of plankton dynamics is of great ecological importance, both because plankton form an important component of the global carbon cycle and couples to the global climate system and because plankton provide the base of the aquatic food chain and therefore drives the productivity of our lakes and oceans. In spite of enormous progress in plankton modelling, there is still a lack of fundamental understanding of even some rather striking phenomena. We address this in this paper with a novel conceptual plankton model that for the first time gives analytical results that simultaneously describes both the within-species cell size distribution and the across-species distribution of plankton biomass.

One of the most remarkable patterns in ecology manifests itself in the distribution of biomass as a function of body size in aquatic ecosystems (Sheldon et al. 1972). Very approximately, equal intervals of the logarithm of body mass contain equal amounts of biomass per unit volume. This implies that biomass density decreases approximately as the inverse of body mass. *Size spectra* with this approximate shape are observed over many orders of magnitude, encompassing both unicellular and multicellular organisms (Gaedke 1992; Quiñones et al. 2003; San Martin et al. 2006) and it has been conjectured that this relationship applies all the way from bacteria to whales (Sheldon et al. 1972). Accordingly, aquatic environments are more populated by small organisms than larger ones in a predictable way (Sheldon and Kerr 1972).

Early theories, without dynamics, gave results consistent with this power law (Platt and Denman 1977) and they were followed by dynamic theories for multicellular organisms (size-spectrum models), where the biomass distribution is an outcome of the processes and interactions between these organisms at different sizes (Silvert and Platt 1978, 1980; Camacho and Solé 2001; Benoît and Rochet 2004; Andersen and Beyer 2006; Capitán and Delius 2010; Datta et al. 2010, 2011; Hartvig et al. 2011; Maury and Poggiale 2013). In these models, multicellular organisms grow by feeding on and killing smaller organisms, thereby coupling the two opposing faces of predation: death of the prey, and body growth of the predator—during which survivors can grow over orders of magnitude. A common feature of the models is the allometric scaling of the rates of the different processes. For recent reviews of size-spectrum modelling see Sprules et al. (2016) and Guiet et al. (2016).

Current models of size-spectrum dynamics are constructed with multicellular, heterotrophic organisms in mind, and make simplifying assumptions about the unicellular plankton on which they ultimately depend to provide a closure for the models (e.g. Hartvig et al. 2011; Datta et al. 2010). The unicellular-multicellular distinction is important. Unicellular plankton encompass autotrophs (phytoplankton) that use inorganic nutrients and light to synthesize their own food, as well as heterotrophs (zooplankton) that feed on other organisms, and mixotrophs that do both. Also, unicellular organisms just double in size before splitting into two roughly equally-sized cells, rather than going through the prolonged somatic growth of multicellular organisms. Since cell masses of unicellular plankton span an overall range of approximately $10^8$, the power law cannot therefore be generated without coexistence of many species.

Coexistence of species in the plankton is itself an unresolved problem. In the case of phytoplankton, the problem is known as ‘the paradox of the plankton’, because of the great diversity of phytoplankton taxa, seemingly unconstrained by the small number of resources they compete for (Hutchinson 1961). There is no consensus yet as to what mechanism(s) can allow a large number of competing species to coexist on a small number of resources (Roy and Chattopadhyay 2007). Hutchinson thought environmental fluctuations could be the answer, but this is currently acknowledged to be insufficient as an explanation (Fox 2013). One promising proposal is a strategy of “killing the winner” that involves a trade-off between competitive ability and defence against enemies (Thingstad and Lignell 1997; Winter et al. 2010) and that resembles the mechanism of predator-mediated coexistence observed in ecology (Leibold 1996; Våge et al. 2014).

In this paper we propose a dynamic trait-based size-spectrum model for plankton that incorporates specific cellular mechanisms for growth, feeding, and reproduction, along with their allometric laws, in order to capture the size spectrum of biomass distribution in this size region of the aquatic ecosystem (Sect. 2). We build on well established models of the cell cycle (Fredrickson et al. 1967; Diekmann et al. 1983; Heijmans 1984; Henson 2003; Friedlander and Brenner 2008; Giometto et al. 2013) but extend them to allow for many coexisting species. The resulting model describes the dynamics of an ecosystem made of a continuum of phytoplankton species living on a single resource, plus a continuum of zooplankton species that feed on smaller cells. For the allometric scaling of the growth and division rate we make use of recent experimental measurements on phytoplankton production (Marañón et al. 2013).

The model is presented in two flavours: an idealised version (Sect. 3) describing cells that grow until exactly doubling their size and then split into two identical cells, and a more general model (Sect. 4) in which cells are allowed to divide in a range of sizes and produce two daughter cells of slightly different sizes. In both cases we provide analytic expressions for the abundance distribution as a function of size for any species.

For both flavours of the model we first study the conditions under which the steady state allows for the coexistence of a continuum of infinitely many phytoplankton species and find—not surprisingly—that a sufficient condition is a death rate that scales allometrically with the same exponent as the growth rate. Then we introduce zooplankton that predate on smaller cells (whether phyto- or zooplankton) and show that predation produces the required scaling of the death rate if, and only if, the whole plankton community conforms to Sheldon’s power law size spectrum with an exponent very close to the observed one. This power law size spectrum arises as the steady state solution in our model (Sect. 5).

In other words, within the model assumptions, coexistence of a continuum of plankton species implies a specific allometric scaling of the death rate and the zooplankton growth rate; the latter allometric scalings imply that the whole community distributes as a power-law in size; and a power-law size distribution of the community implies the coexistence of a continuum of plankton species. This is the main result of our work. It reveals that the paradox of the plankton and the observed size spectrum in aquatic ecosystems are but two manifestations of the same phenomenon, and are both deeply rooted in the allometric scaling of basic physiological rates. In Sect. 6 we show that this allometric scaling makes the model invariant under scale transformations. This provides another formulation of our explanation for the origin of the Sheldon spectrum.

## Size- and species-resolved phytoplankton model

Our model for phytoplankton is a multispecies variant of the population balance equation (PBE) model (Fredrickson et al. 1967; Henson 2003; Friedlander and Brenner 2008). Phytoplankton are assumed to be made mostly of unicellular autotrophs that grow through the absorption of inorganic nutrients from the environment and eventually split into two roughly equal-size daughter cells.

Cells will be described by their current size *w* and by a size $w_*$ characteristic of the cell’s species. For this characteristic size we choose the maximum size a cell can reach. We measure sizes with respect to some reference size, so that *w* and $w_*$ are dimensionless. This will avoid strange fractional dimensions that would otherwise arise in allometric scaling expressions later.

The two basic processes of the cellular dynamics are growth and division. We describe these in detail in the following subsections before using them in Sect. 2.3 to give the dynamical population balance equation for phytoplankton abundances.

### Cell growth

A widely accepted model for organismal growth was proposed long ago by von Bertalanffy (1957). Although originally it was devised for multicellular organisms, it has recently been argued that a similar model can be used to describe the growth of microorganisms (Kempes et al. 2011). According to von Bertalanffy’s model, the rate at which an organism grows is the result of a competition between the gain of mass through nutrient uptake and its loss through metabolic consumption. Both terms exhibit allometric scaling, thus

2.1 $$\begin{aligned} \frac{dw}{dt}=Aw^{\alpha }-Bw^{\beta }. \end{aligned}$$

A typical assumption is $\alpha =2/3$ (nutrient uptake occurs through the organismal membrane) and $\beta =1$ (metabolic consumption is proportional to body mass, Kempes et al. 2011). However other choices are possible and different values have been empirically obtained (Law et al. 2016). Whichever the values, it seems reasonable to constrain the exponents to satisfy $\alpha <\beta$—leading to a slow-down of growth as cells get very large. Constants *A* and *B* will vary from species to species, so depend on $w_*$.

With this model we can calculate the doubling period of a cell, defined as the time $T(w_*)$ it takes to grow from $w_*/2$ to $w_*$:

2.2 $$\begin{aligned} T(w_*)=\int _{w_*/2}^{w_*}\frac{dw}{Aw^{\alpha }-Bw^{\beta }} =w_*\int _{1/2}^1\frac{du}{Aw_*^{\alpha }u^{\alpha }-Bw_*^{\beta }u^{\beta }}, \end{aligned}$$

where $$u=w/w_*$$.

It turns out that this doubling period has been experimentally measured for many different species of phytoplankton under the same environmental conditions. All the results for phytoplankton cells larger than $\sim$5 $\upmu$m seem to scale with the same function $T=\tau w_*^{\xi }$, where $\tau$ is a species-independent constant. Cells smaller than $\sim$5 $\upmu$m have a doubling period which increases, rather than decreases, as they become smaller (Marañón et al. 2013). To all purposes then, our model will describe the community spectrum from $\sim$5 $\upmu \hbox {m}$ upward. There is some controversy in the experimental literature about the right value of the exponent $\xi$ (Law et al. 2016), but we need not be concerned by it. When we need a concrete value we will adopt the most recent value $\xi \approx 0.15$ (Marañón et al. 2013).

The allometric scaling observed for the duplication period can only be compatible with Eq. (2.2) provided

2.3 $$\begin{aligned} A\equiv aw_*^{1-\alpha -\xi }, \qquad B\equiv bw_*^{1-\beta -\xi }, \end{aligned}$$

where *a* and *b* do not depend on $w_*$. Then the proportionality constant $\tau$ is given by

2.4 $$\begin{aligned} \tau =\int _{1/2}^1\frac{du}{au^{\alpha }-bu^{\beta }}. \end{aligned}$$

Since $\tau$, $\alpha$, and $\beta$ can be experimentally determined, this equation imposes a constraint on the constants *a* and *b*.

In summary, joining a von Bertalanffy model for the growth rate with the experimental observations for the division rate yields the growth model

2.5 $$\begin{aligned} \frac{dw}{dt}=G_p(w,w_*) =w_*^{1-\xi }\left[ a\left( \frac{w}{w_*}\right) ^{\alpha } -b\left( \frac{w}{w_*}\right) ^{\beta }\right] . \end{aligned}$$

It is worth noting that this growth rate is a homogeneous function satisfying

2.6 $$\begin{aligned} G_p(\lambda w,\lambda w_*)=\lambda ^{1-\xi }G_p(w,w_*) \end{aligned}$$

for any $\lambda >0$. Also notice that $a>b$ guarantees $G_p(w,w_*)>0$ for all $0\leqslant w\leqslant w_*$.

### Cell division

Let $K(w,w_*)$ denote the division rate of a cell of current size *w* and maximum size $w_*$. We expect $K(w,w_*)$ to grow sharply near $w=w_*$—to ensure that division is guaranteed to occur before a cell reaches its maximum size. A widely studied cell division mechanism assumes a ‘sloppy size control’ of the cell division cycle (Powell 1964; Tyson and Diekmann 1986). Essentially, this means that cells can duplicate at any moment after reaching a threshold size $w_\text {th}$ and before reaching their largest possible size $w_*$. By proposing a suitable function $K(w,w_*)$ Tyson and Diekmann (1986) were able to fit the size distribution at division of a yeast.

While Tyson and Diekmann (1986) assumed that duplication produces two equally-sized daughter cells, we will in Sect. 4 allow the size of the daughter cells to be described by $Q(w|w')$, the probability density that a cell of size $w'$ splits into two cells of sizes *w* and $w'-w$. By construction $Q(w|w')=0$ if $w\geqslant w'$ or $w\leqslant 0$, it bears the symmetry $Q(w'-w|w')=Q(w|w')$ and satisfies the normalising condition

2.7 $$\begin{aligned} \int _0^{\infty }Q(w|w')\,dw=1 \end{aligned}$$

for all $$0<w'<\infty $$.

It is reasonable to assume that $Q(w|w')$ is peaked around $w=w'/2$—daughter cells will be roughly half the size of the parent cell. Another reasonable assumption is that this distribution scales with cell size (i.e., fluctuations around the ideal splitting size $w=w'/2$ are relative to $w'$). This amounts to assuming that $Q(w|w')$ is a homogeneous function of *w* and $w'$,

2.8 $$\begin{aligned} Q(\lambda w,\lambda w')=\lambda ^{-1}Q(w,w'). \end{aligned}$$

The scaling exponent of $-1$ is due to the fact that *Q* is a probability density. We can therefore write *Q* in the scaling form

2.9 $$\begin{aligned} Q(w|w')=\frac{1}{w'}\,q\left( \frac{w}{w'}\right) , \qquad \text { where }\int _0^{\infty }q(x)\,dx=1. \end{aligned}$$

### Cell population dynamics

We will assume that the number of species and their population is large enough so that we can make a continuum description through a density function $p(w,w_*,t)$, such that $p(w,w_*,t)\,dwdw_*$ is the number of cells per unit volume whose maximum sizes are between $w_*$ and $w_*+dw_*$ and whose sizes at time *t* are between *w* and $w+dw$.

With these ingredients, the time evolution of the abundances $p(w,w_*,t)$ will be given by the population balance equation (PBE) (Fredrickson et al. 1967; Henson 2003; Friedlander and Brenner 2008)

2.10 $$\begin{aligned} \begin{aligned} \frac{\partial }{\partial t}p(w,w_*,t) =&\, -\frac{\partial }{\partial w}\big [G_p(w,w_*)p(w,w_*,t)\big ] \\&+\,2\int _0^{w_*}Q(w|w')K(w',w_*)p(w',w_*,t)\,dw' \\&-\,K(w,w_*)p(w,w_*,t)-M(w,w_*)p(w,w_*,t). \end{aligned} \end{aligned}$$

The first two terms describe the dynamics of a growing organism as an extension of the McKendrick–von Foerster equation (Silvert and Platt 1978, 1980). The third term is the rate at which cells of size *w* are produced from the division of cells of size $0<w'<w_*$—the factor 2 taking care of the fact that each parent cell yields two daughter cells. The fourth term is the rate at which cells of size *w* divide. The last term is the rate at which cells of size *w* die for whatever reason. The same equation describes this process for any species, hence $w_*$ enters as a parameter in every rate function involved.

The fact that every negative term on the right hand side of the population balance equation (2.10) is proportional to $p(w,w_*,t)$, ensures the necessary property that the population density $p(w,w_*,t)$ can never evolve to be negative.

### Nutrient dynamics

The growth model just developed assumes an infinite abundance of nutrients. In real aquatic ecosystems nutrients are limited though, and growth is hindered when nutrients are scarce. Accordingly, we need to modify our growth model in order to take limited nutrients into account.

In the von Bertalanffy equation (2.5) for the cell growth rate, the first term describes the nutrient uptake through the cell membrane, and it is modulated by the rate *a*. This rate will of course depend on the availability of the nutrients needed for growth. Denoting by $N$ the amount of nutrient per unit volume, we need to replace *a* by a function $a(N)$. The simplest way to do this is through the Monod equation (Herbert et al. 1956)

2.11 $$\begin{aligned} a(N)=a_{\infty }\frac{N}{r+N}, \end{aligned}$$

with $r$ the Michaelis–Mertens constant. This function has the important property that the factor $a(N)$ monotonically increases from 0 toward its saturation value $a_{\infty }$. However, other choices for $a(N)$ with this property are also possible.

Likewise, the details of how the nutrient dynamics is modelled are not important for our conclusions. All we will require is that the uptake of nutrient by the plankton leads to a corresponding depletion in the nutrient $N$. Also, in order to sustain a non-zero plankton population, there needs to be some replenishment of nutrient. The PBE model incorporates that through a chemostat of maximum capacity $N_0$ (Fredrickson et al. 1967; Heijmans 1984; Henson 2003):

2.12 $$\begin{aligned} \frac{dN}{dt}=\varrho (N)-\sigma (N,p), \qquad \varrho (N)=\varrho _0\left( 1-\frac{N}{N_0}\right) . \end{aligned}$$

Here $\sigma (N,p)$ represents the rate of nutrients consumption by all phytoplankton cells, which is proportional to the uptake rate [the positive term in the expression for $G_p(w,w_*,t)$ in Eq. (2.5)], integrated over all species sizes $w_*$ and all cell sizes *w*:

2.13 $$\begin{aligned} \sigma (N,p)=\frac{a(N)}{\theta }\int _0^{\infty }dw_*\,w_*^{1-\alpha -\xi } \int _0^{w_*}dw\,w^{\alpha }p(w,w_*,t). \end{aligned}$$

The proportionality constant $\theta$ is the *yield* constant, i.e. the amount of biomass generated per unit of nutrient.

## Idealised cell division process

The important features of our model are insensitive to the details of the cell division process. So it makes sense to first exhibit these features by solving the model with the simplest idealised version of the cell division. Thus in this section we assume that cells only split when they reach exactly the size $w_*$, and they generate two identically sized daughter cells (Diekmann et al. 1983).

This idealised cell division has an undesirable property: Consider a peak in abundance around a particular size. Due to growth of the cells making up the peak, it will move through the size spectrum without changing its shape until it reaches the maximum size $w_*$. There all cells will divide to produce daughter cells at exactly the size $w_*/2$, producing a new peak again of the same shape. This peak will then again move up to $w_*$, divide and restart its journey, ad infinitum. In short: the solutions in this idealised model will be periodic, rather than approaching the steady state solution. This will be remedied in the general case that we will discuss in Sect. 4.

### Dynamic equations

The idealised cell division amounts to choosing $Q(w|w')=\delta (w-w'/2)$—two identical daughter cells—and $K(w,w_*)=\kappa (w_*)\delta (w-w_*)$—division occurs only when $w=w_*$. Here $\delta (x)$ denotes the Dirac delta function. The parameter $\kappa (w_*)$ will be determined below. This choice transforms the evolution equation (2.10) into

3.1 $$\begin{aligned} \begin{aligned} \frac{\partial }{\partial t}p(w,w_*,t) =&\, -\frac{\partial }{\partial w}\big [G_p(w,w_*)p(w,w_*,t)\big ] \\&+\kappa (w_*)p(w_*,w_*,t)[2\delta (w-w_*/2)-\delta (w-w_*)] \\&-M(w,w_*)p(w,w_*,t), \end{aligned} \end{aligned}$$

and of course $p(w,w_*,t)=0$ for $w>w_*$ and $w<w_*/2$.

The two delta functions on the right-hand side of Eq. (3.1) imply that the function $p(w,w_*,t)$ must be discontinuous at $w=w_*/2$ and $w=w_*$ (recall that $\varTheta '(x)=\delta (x)$, where $\varTheta (x)$ is a Heaviside step function, equal to 1 for $x>0$ and to 0 for $x<0$). The height of the two discontinuities must be such that the derivative of the right-hand side cancels the two deltas. This leads to the two conditions

3.2 $$\begin{aligned} G_p(w_*,w_*)p(w_*,w_*,t)&=\kappa (w_*)p(w_*,w_*,t), \end{aligned}$$

3.3 $$\begin{aligned} G_p(w_*/2,w_*)p(w_*/2,w_*,t)&=2\kappa (w_*)p(w_*,w_*,t). \end{aligned}$$

Equation (3.2) determines $\kappa (w_*)=G_p(w_*,w_*)$, so that Eq. (3.3) implies the boundary condition

3.4 $$\begin{aligned} G_p(w_*/2,w_*)p(w_*/2,w_*,t)=2G_p(w_*,w_*)p(w_*,w_*,t). \end{aligned}$$

Notice that, since $\delta (\lambda w-\lambda w_*)=\lambda ^{-1}\delta (w-w_*)$, this link between the division rate function $K(w,w_*)$ and the growth rate $G_p(w,w_*)$ renders the former homogeneous in its arguments,

3.5 $$\begin{aligned} K(\lambda w,\lambda w_*) =\lambda ^{-\xi }K(w,w_*). \end{aligned}$$

In summary, when considering the idealised division process, the phytoplankton density $p(w,w_*,t)$ is described by the equation

3.6 $$\begin{aligned} \frac{\partial }{\partial t}p(w,w_*,t)+ \frac{\partial }{\partial w}\big [G_p(w,w_*)p(w,w_*,t)\big ]+ M(w,w_*)p(w,w_*,t)=0, \end{aligned}$$

in the interval $w_*/2\leqslant w\leqslant w_*$, with the boundary condition (3.4). This is coupled to Eqs. (2.12) and (2.13) for the nutrient.

### Steady state

We can look for solutions of Eq. (3.6) that do not depend on time by solving the first order ordinary differential equation

3.7 $$\begin{aligned} \frac{\partial }{\partial w}\big [G_p(w,w_*)p(w,w_*)\big ]+ M(w,w_*)p(w,w_*)=0, \qquad \frac{w_*}{2}\leqslant w\leqslant w_*, \end{aligned}$$

with the boundary condition (3.4). A straightforward integration of Eq. (3.7) yields

3.8 $$\begin{aligned} p(w,w_*)=p(w_*,w_*)\frac{G_p(w_*,w_*)}{G_p(w,w_*)}\exp \left\{ \int _{w}^{w_*}\frac{M(w',w_*)}{G_p(w',w_*)}\,dw'\right\} , \end{aligned}$$

where $p(w_*,w_*)$ is some (as yet) arbitrary value. If we now impose the boundary condition  (3.4) on the solution (3.8) we arrive at the condition

3.9 $$\begin{aligned} \int _{w_*/2}^{w_*}\frac{M(w',w_*)}{G_p(w',w_*)}\,dw'=\log 2. \end{aligned}$$

The left-hand side of this condition is in general a function of $w_*$. This means that only those species whose maximum sizes are such that Eq. (3.9) holds can have a non-zero stationary abundance. The only possibility for the remaining species is $p(w_*,w_*)=0$, i.e., extinction.

There is only one case in which Eq. (3.9) can hold for *all* species, namely when the death rate is a homogeneous function $M(\lambda w,\lambda w_*)=\lambda ^{-\xi }M(w,w_*)$, or, equivalently, if it has the shape

3.10 $$\begin{aligned} M(w,w_*)=w_*^{-\xi }m(w/w_*) \end{aligned}$$

for some function $m(x)$. This allometric scaling of the death rate is a necessary condition for coexistence. It is also a sufficient condition, because provided this condition is met, the solution (3.8) takes the explicit form

3.11 $$\begin{aligned} p(w,w_*)=p(w_*,w_*)\phi (w/w_*), \end{aligned}$$

with

3.12 $$\begin{aligned} \phi (x)= \frac{a(N)-b}{a(N)x^{\alpha }-bx^{\beta }} \exp \left\{ \int _x^1\frac{m(y)}{a(N)y^{\alpha }-b\,y^{\beta }}\,dy\right\} . \end{aligned}$$

In other words, all species show the same size distribution up to a constant $p(w_*,w_*)$ that determines the overall abundance of that species.

In this case the boundary condition (3.9) becomes

3.13 $$\begin{aligned} \int _{1/2}^{1}\frac{m(x)}{a(N)x^{\alpha }-bx^{\beta }}\,dx=\log 2. \end{aligned}$$

This equation holds for one and only one value of $N$ (remember that $a(N)$ is an increasing function of $N$ and $a(0)=0$ and $a_{\infty }>b$). For any value other than this, no steady state solution is possible except full extinction. On the other hand, for this specific $N$ all species *coexist* in the steady state.

According to Eq. (2.12), the condition for $N$ to be the nutrient level at the steady state is $\varrho (N)=\sigma (N,p)$. Using the expression (3.11) for the steady-state $p(w,w_*)$ in the expression (2.13) for $\sigma (N,p)$, this can be expressed as the following constraint on the overall abundances:

3.14 $$\begin{aligned} \int _0^{\infty }w_*^{2-\xi }p(w_*,w_*)\,dw_*= \frac{\theta \varrho (N)}{a(N)} \left( \int _0^1x^{\alpha }\phi (x)\,dx\right) ^{-1}. \end{aligned}$$

This is only a single linear constraint on the function $p(w_*,w_*)$ and thus is far from determining it uniquely.

To summarise this section: if the death rate scales allometrically with size and all phytoplankton species share a common limited resource then there is a steady state of the system in which all species coexist on this single resource. The resource level is tuned by consumption. In its turn, its value imposes a global constraint on the abundances of phytoplankton species.

This result is a manifestation of the ‘paradox of the plankton’ (Hutchinson 1961), and reveals a mechanism by which it might come about: a similar allometric scaling for both the growth and the death rate. As of now, it is hard to think of a reason why this similar scaling should occur, but we will return to this point in Sect. 5 where we will show that predation is one possible mechanism.

## General division process

Although the idealised division process described in the previous section is a simple setup that provides important insights on the system behaviour, it has some undesirable features that call for improvements. Perhaps the worst of them is the fact that we illustrated at the start of Sect. 3: any irregularity of the initial distribution of cell sizes will remain there forever because there is nothing that smooths it out. Consequently, the distribution could never evolve towards the steady-state distribution. Two mechanisms can achieve the necessary size mixing to provide this smoothing: first, the fact that cells do not split only when they exactly reach the size $w_*$, and second, the fact that the sizes of the two daughter cells are not identical. Both of them require introducing functions $K(w,w_*)$ and $Q(w|w')$ more general than Dirac’s deltas.

### Model constraints

The problem boils down to solving the PBE (2.10). Although linear, this is a difficult integro-differential problem whose general solution can only be obtained in the form of an infinite functional series (Heijmans 1984). This notwithstanding, there is a general class of functions $K(w,w_*)$ and $Q(w|w')$ for which a closed form solution is possible, and the constraints that define this class are general enough to describe real situations. Let us spell out these constraints.

To guarantee that all cells divide before growing beyond size $w_*$ the rate $K(w,w_*)$ is chosen to satisfy

4.1 $$\begin{aligned} \int _0^{w_*}K(w,w_*)dw=\infty . \end{aligned}$$

There will be some smallest size $w_\text {th}$ below which cells can not divide. Hence $K(w,w_*)$ is non-zero only for $w_\text {th}<w<w_*$. Let us also assume that $Q(w|w')$ is non-zero only for $(1-\delta )w'/2<w<(1+\delta )w'/2$ for some $\delta$ that measures the maximum variability of the daughter cells’ sizes relative to the parent’s. With these two assumptions it is clear that the largest possible size of a daughter cell is $w_+=(1+\delta )w_*/2$. Like (Powell 1964) we further assume $w_+<w_\text {th}$.

Let us split the abundance into ‘large’ and ‘small’ cells according to

4.2 $$\begin{aligned} p(w,w_*,t)= {\left\{ \begin{array}{ll} p_l(w,w_*,t), &{}\quad w\ge w_+, \\ p_s(w,w_*,t), &{}\quad w\le w_+. \end{array}\right. } \end{aligned}$$

Then the integral term in the right-hand side of Eq. (2.10) will make no contribution for any $w>w_+$, and we will have, for $w_+\leqslant w\leqslant w_*$,

4.3 $$\begin{aligned} \begin{aligned} \frac{\partial }{\partial t}p_l(w,w_*,t) =&\, -\frac{\partial }{\partial w}\big [G_p(w,w_*)p_l(w,w_*,t)\big ] \\&-K(w,w_*)p_l(w,w_*,t)-M(w,w_*)p_l(w,w_*,t). \end{aligned} \end{aligned}$$

Due to our assumption that $w_+<w_\text {th}$ we can replace $p(w,w_*,t)$ by $p_l(w,w_*,t)$ in the integral term of Eq. (2.10); hence, for $0\leqslant w\leqslant w_+$,

4.4 $$\begin{aligned} \begin{aligned} \frac{\partial }{\partial t}p_s(w,w_*,t) =&\, -\frac{\partial }{\partial w}\big [G_p(w,w_*)p_s(w,w_*,t)\big ] \\&+2\int _{w_\text {th}}^{w_*}Q(w|w')K(w',w_*)p_l(w',w_*,t)\,dw' \\&-M(w,w_*)p_s(w,w_*,t). \end{aligned} \end{aligned}$$

We have transformed the original problem into two, each in a different interval. The first problem, Eq. (4.3), is a homogeneous linear differential equation decoupled from the second one, Eq. (4.4), which turns out to be—once the solution of the first problem is known—a non-homogeneous linear differential equation.

These two equations, (4.3) and (4.4), have to be supplemented with the boundary conditions

4.5 $$\begin{aligned} p_s(0,w_*,t)=0,~~~~ p_s(w_+,w_*,t)=p_l(w_+,w_*,t),~~~~ p_l(w_*,w_*,t) =0. \end{aligned}$$

### Scaling behaviour of the division rate

In the idealised model (Sect. 3.1), since $K(w,w_*)$ was proportional to a Dirac’s delta, we could obtain its scaling from that of $G_p(w,w_*)$ straight away. Unfortunately, the argument is no longer valid for this more general setup. There is a workaround though: we can prove that $K(w,w_*)$ scales as in the idealised case from the empirical observation that the population growth rate of a single species in a nutrient-rich environment scales as $\varLambda \sim w_*^{-\xi }$ (Marañón et al. 2013).

Suppose we prepare a nutrient-rich culture of cells of maximum size $w_*$. Equations (4.3) and (4.4) will describe the abundances at different sizes. In this situation, for some initial time interval we can assume $M(w,w_*)=0$, so the population will increase exponentially at rate $\varLambda$. Introducing $p_l(w_+,w_*,t)=p_l(w_+,w_*)e^{\varLambda t}$ and $p_s(w_+,w_*,t)=p_s(w_+,w_*)e^{\varLambda t}$ into those equations we end up with

4.6 $$\begin{aligned} \frac{\partial }{\partial w}\big [G_p(w,w_*)p_l(w,w_*)\big ] =&\, -K(w,w_*)p_l(w,w_*)-\varLambda p_l(w,w_*), \end{aligned}$$

4.7 $$\begin{aligned} \frac{\partial }{\partial w}\big [G_p(w,w_*)p_s(w,w_*)\big ] =&\, 2\int _{w_\text {th}}^{w_*}Q(w|w')K(w',w_*)p_l(w',w_*)\,dw' \nonumber \\&-\varLambda p_s(w,w_*). \end{aligned}$$

The solution of Eq. (4.6) is

4.8 $$\begin{aligned} p_l(w,w_*)&=p_l(w_+,w_*)\frac{G_p(w_+,w_*)}{G_p(w,w_*)}E(w,w_*), \end{aligned}$$

4.9 $$\begin{aligned} E(w,w_*)&=\exp \left\{ -\int _{w_+}^w\frac{K(w',w_*)+\varLambda }{G_p(w',w_*)}\,dw'\right\} , \end{aligned}$$

with $p_l(w_+,w_*)$ an undetermined constant.

As for Eq. (4.7), its solution is

4.10 $$\begin{aligned} p_s(w,w_*)&=p_l(w,w_*) \left[ 1-\int _w^{w_+}\frac{H(w',w_*)}{E(w',w_*)}\,dw'\right] , \end{aligned}$$

4.11 $$\begin{aligned} H(w,w_*)&=2\int _{w_\text {th}}^{w_*}Q(w|w')\frac{K(w',w_*)}{G_p(w',w_*)} E(w',w_*)\,dw'. \end{aligned}$$

The condition $p_l(w_+,w_*,t)=p_s(w_+,w_*,t)$ is already met, and the boundary condition $p_l(w_*,w_*,t)=0$ follows from Eq. (4.1). The boundary condition $p_s(0,w_*,t)=0$ implies

4.12 $$\begin{aligned} \int _0^{w_+}\frac{H(w',w_*)}{E(w',w_*)}\,dw'=1. \end{aligned}$$

This equation determines the population growth rate $\varLambda$ and allows us to rewrite Eq. (4.10) as

4.13 $$\begin{aligned} p_s(w,w_*)=p_l(w,w_*)\int _0^w\frac{H(w',w_*)}{E(w',w_*)}\,dw'. \end{aligned}$$

Equation (4.12) is the key to infer the scaling of $K(w,w_*)$. If, in agreement with empirical measurements, $\varLambda =\ell w_*^{-\xi }$ with $\ell$ independent on $w_*$, then Eq. (4.12) becomes

$$\begin{aligned} 2\int _0^{\frac{1+\delta }{2}}dx\int _{\frac{w_{\text {th}}}{w_*}}^1\frac{dy}{y}\, q\left( \frac{x}{y}\right) \frac{w_*^{\xi }K(w_*y,w_*)}{a(N)y^{\alpha }-by^{\beta }}\exp \left\{ \int _y^x \frac{w_*^{\xi }K(w_*z,w_*)+\ell }{a(N)z^{\alpha }-bz^{\beta }}\,dz\right\} =1, \end{aligned}$$

a condition that can only be met provided $w_{\text {th}}/w_*$ does not depend on $w_*$ and

4.14 $$\begin{aligned} K(w,w_*)=w_*^{-\xi }k(w/w_*), \end{aligned}$$

in other words, if the scaling $K(\lambda w,\lambda w_*)= \lambda ^{-\xi }K(w,w_*)$ holds. Of course it is also intuitively clear that the division rate has to scale as $w_*^{-\xi }$ given that the doubling period $T(w_*)$ scales as $w_*^\xi$, as discussed in Sect. 2.1. Thus we see that the same empirical observation that leads to the functional form (2.5) for $G_p(w,w_*)$ also leads to Eq. (4.14).

### Steady state

The steady state of Eqs. (4.3) and (4.4) is readily obtained by replacing $\varLambda$ with $M(w,w_*)$ in Eqs. (4.6) and (4.7). The solution will be as given by Eqs. (4.8) and (4.13), but with $E(w,w_*)$ given by

4.15 $$\begin{aligned} E(w,w_*)=\exp \left\{ -\int _{w_+}^w\frac{K(w',w_*)+M(w',w_*)}{G_p(w',w_*)}\,dw'\right\} . \end{aligned}$$

The boundary condition (4.12) now fixes the value of $a(N)$ in the function $G_p(w,w_*)$ and thereby determines the steady-state nutrient level $N$.

The same considerations as for the idealised case hold here. Equation (4.12) will, in general, depend on $w_*$ and therefore hold for at most one or a few species. The other species are extinct in the steady state. Given the scaling (4.14) for the division rate, the requirement for coexistence of all species is the scaling (3.10) of the death rate, because then $E(w,w_*)=e(w/w_*)$ and $H(w,w_*)=w_*^{-1}h(w/w_*)$, where

4.16 $$\begin{aligned} e(x)&=\exp \left\{ -\int _{\frac{1+\delta }{2}}^x \frac{k(y)+m(y)}{a(N)y^{\alpha }-by^{\beta }}\,dy\right\} , \end{aligned}$$

4.17 $$\begin{aligned} h(x)&=2\int _{\frac{w_\text {th}}{w_*}}^1\frac{k(y)e(y)}{a(N)y^{\alpha } -by^{\beta }}q\left( \frac{x}{y}\right) \frac{1}{y}\,dy, \end{aligned}$$

and the boundary condition (4.12) becomes

4.18 $$\begin{aligned} \int _0^{\frac{1+\delta }{2}}\frac{h(x)}{e(x)}\,dx=1 \end{aligned}$$

regardless of the species.

Finally, the steady state abundances are given by

4.19 $$\begin{aligned} p(w,w_*)=p(w_+,w_*)\psi (w/w_*), \end{aligned}$$

where $p(w_+,w_*)$ is an undetermined function of $w_*$ and

4.20 $$\begin{aligned} \psi (x)&=\frac{a(N)\left( \frac{1+\delta }{2}\right) ^{\alpha }- b\left( \frac{1+\delta }{2}\right) ^{\beta }}{a(N)x^{\alpha }- bx^{\beta }}e(x)\varTheta (x), \end{aligned}$$

4.21 $$\begin{aligned} \varTheta (x)&= {\left\{ \begin{array}{ll} 1, &{}\quad x>\frac{1+\delta }{2}, \\ \displaystyle \int _0^x\frac{h(y)}{e(y)}\,dy, &{}\quad x<\frac{1+\delta }{2}. \end{array}\right. } \end{aligned}$$

**Fig. 1:**
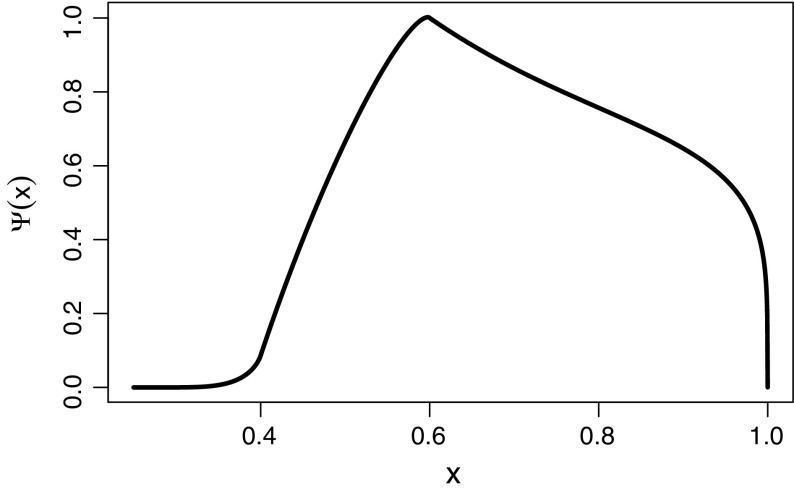
The steady-state within-species size-distribution $\psi (x)$, with constant mortality, growth parameter values $a=0.7, b=0.5, \alpha =0.85, \beta =1$, a division threshold of $0.7w_*$ and rate $K(w,w_*)$ given by Eq. (4.14) with $k(x)=4(x-0.7)^2/(1-x)$ and daughter cell sizes distributed uniformly between $0.4w_*$ and $0.6w_*$

A few remarks will make clear what the abundance distribution looks like. To begin with, property (4.1) of $K(w,w_*)$ implies that $e(1)=0$, so $p(w_*,w_*)=0$. On the other hand, given that $q(x/y)=0$ except for $(1-\delta )/2<x/y<(1+\delta )/2$ (i.e. $2x/(1+\delta )<y<2x/(1-\delta )$), function $h(x)=0$ except for $w_\text {th}(1-\delta )/2w_*<x<(1+\delta )/2$. This means that $p(w,w_*)=0$ for all $w\leqslant w_\text {th}(1-\delta )/2$ and that it is a differentiable function in the whole interval $[0,w_*]$. From the fact that $\partial p(w,w_*)/\partial w<0$ when $w>w_+$ we can conclude that the maximum of this function will occur at some point $w_{\text {max}}<w_+$ (Fig. 1).

## Predation by zooplankton

In the idealised model of cell division of Sect. 3 as well as in the more general model of Sect. 4, we have seen that the allometric scaling of the death rate is a crucial ingredient to the coexistence of multiple phytoplankton species living on one or a few resources. The main cause of phytoplankton death is predation. Many species feed on phytoplankton, from unicellular organisms to whales. Even though a detailed model of the marine ecosystem would have to include these very many types of grazers as well as their predators, in order to keep the model simple—and at the same time to illustrate how predation can provide the sort of death rate necessary for coexistence—we will focus only on unicellular zooplankton.

We will denote the density of zooplankton cells by $z(w,w_*,t)$, so that the number of cells in a unit volume with a maximum size between $w_*$ and $w_*+dw_*$ that at time *t* have a size between *w* and $w+dw$ is $z(w,w_*,t)dwdw_*$.

To model predation, we introduce a new rate function $S(w,w')$: the rate at which a given predator cell of size *w* preys on a given prey cell of size $w'$. This rate could also be allowed to depend on the specific predator and prey species through $w_*$ and $w_*'$. However, this would introduce unnecessary notational complexity without adding anything qualitatively different to the discussion.

A common ansatz for this rate function in the literature is

5.1 $$\begin{aligned} S(w,w')=w^{\nu }s(w/w'). \end{aligned}$$

The second factor is a kernel that selects the preferred prey size relative to the size of the predator (Wirtz 2012). The power of *w* in front of it arises from the foraging strategy, which is known to depend allometrically on cell size (DeLong and Vasseur 2012).

The mortality rate due to predation is obtained by integrating the contributions from all predators. For the sake of completeness, a background death due to other sources—for which we will adopt the allometric scaling (3.10)—will be added. Thus we set

5.2 $$\begin{aligned} M(w,w_*,t)=\int _0^\infty S(w',w)z_c(w',t)\,dw'+ w_*^{-\xi }m_b(w/w_*), \end{aligned}$$

where the zooplankton community spectrum is defined as

5.3 $$\begin{aligned} z_c(w,t)=\int _0^\infty z(w,w_*,t)\,dw_*. \end{aligned}$$

Zooplankton abundance is described by an equation similar to Eq. (2.10),

5.4 $$\begin{aligned} \begin{aligned} \frac{\partial }{\partial t}z(w,w_*,t) =&\, -\frac{\partial }{\partial w}\big [G_z(w,w_*,t)z(w,w_*,t)\big ] \\&+2\int _0^{\infty }Q(w|w')K_z(w',w_*,t)z(w',w_*,t)\,dw' \\&-K_z(w,w_*,t)z(w,w_*,t)-M(w,w_*,t)z(w,w_*,t), \end{aligned} \end{aligned}$$

where the growth rate is now

5.5 $$\begin{aligned} G_z(w,w_*,t)=\int _0^\infty S(w,w')\varepsilon w' \left[ p_c(w',t)+z_c(w',t)\right] dw' -bw_*^{1-\xi }\left( \frac{w}{w_*}\right) ^{\beta }, \end{aligned}$$

with the phytoplankton community spectrum defined as

5.6 $$\begin{aligned} p_c(w,t)=\int _0^\infty p(w,w_*,t)\,dw_*. \end{aligned}$$

The first term in (5.5) represents the uptake of nutrients from predation. The factor $\varepsilon$ expresses the efficiency with which prey biomass $w'$ is converted into predator biomass. It is assumed that predators prey indiscriminately on all species of cells, whether zoo- or phytoplankton. The second term accounts for the metabolic consumption. Although we choose this to be the same as for phytoplankton cells [see Eq. (2.5)], substituting different values for *b* and $\beta$ would not change the results of the model qualitatively.

The steady state of the model we have just introduced has an important property that is the main result of this paper, namely that, under the assumptions of the model—in particular the allometric scalings assumed for for the phytoplankton growth rate [Eq. (2.6)] as well as for the predation kernel [Eq. (5.1)], the death rate $M(w,w_*)$ and the zooplankton growth rate $G_z(w,w_*)$ scale allometrically as

5.7 $$\begin{aligned} M(\lambda w,\lambda w_*)=\lambda ^{-\xi }M(w,w_*) ~~\text { and }~~G_z(\lambda w,\lambda w_*)=\lambda ^{1-\xi }G_z(w,w_*) \end{aligned}$$

if, and only if, the community spectra of the phyto- and zooplankton scale as

5.8 $$\begin{aligned} p_c(\lambda w) =\lambda ^{-\gamma }p_c(w)~~\text { and }~~ z_c(\lambda w)=\lambda ^{-\gamma }z_c(w), \end{aligned}$$

with $$\gamma =1+\nu +\xi $$.

The importance of this result lies in the fact that, according to the discussion of Sects. 3.2 [in the paragraph containing Eq. (3.10)] and 4.3 (second paragraph), this allometric scaling of $M(w,w_*)$ is a necessary and sufficient condition for the steady state to exhibit a species-rich phytoplankton community, and similarly, given the scaling of $M(w,w_*)$, that of $G_z(w,w_*)$ becomes then a necessary and sufficient condition for the steady state to exhibit a species-rich zooplankton community. Accordingly, the paradox of the plankton and the power-law size spectrum of the plankton community are *two manifestations of one single phenomenon*—which also expresses itself in the allometric scaling of those two rates.

We will discuss this point further in Sect. 7, and devote the rest of this section to proving this result. If we substitute $z_c=z_0w^{-\gamma }$ within Eq. (5.2) we obtain

5.9 $$\begin{aligned} M(w,w_*)=w_*^{-\xi }m(w/w_*), \quad m(x)=m_b(x)+z_0\,x^{-\xi } \int _0^{\infty }y^{-\xi -1}s(y)\,dy. \end{aligned}$$

This trivially satisfies the required allometric scaling. If we substitute both $p_c=\phi _0w^{-\gamma }$ and $z_c=z_0w^{-\gamma }$ within (5.5) we arrive at

5.10 $$\begin{aligned} \begin{aligned} G_z(w,w_*)&=w_*^{1-\xi }\left[ a_{pz}\left( \frac{w}{w_*}\right) ^{1-\xi } -b\left( \frac{w}{w_*}\right) ^{\beta }\right] , \\ a_{pz}&=\varepsilon (p_0+z_0)\int _0^{\infty } x^{\gamma -3}s(x)\,dx. \end{aligned} \end{aligned}$$

This also complies with the required allometric scaling.

To prove the converse we impose the scaling $M(\lambda w,\lambda w_*)= \lambda ^{-\xi }M(w,w_*)$ on Eq. (5.2), which leads to

$$\begin{aligned} \int _0^{\infty }S(w',\lambda w)z_c(w')\,dw'= \lambda ^{-\xi }\int _0^{\infty }S(w',w)z_c(w')\,dw'. \end{aligned}$$

Changing the variable $w'=\lambda u$ and using the scaling $S(\lambda w, \lambda w')=\lambda ^{\nu }S(w,w')$ derived from (5.1), this equation transforms into

$$\begin{aligned} \lambda ^{1+\nu }\int _0^{\infty }S(u,w)z_c(\lambda u)\,du= \lambda ^{-\xi }\int _0^{\infty }S(w',w)z_c(w')\,dw', \end{aligned}$$

which holds if, and only if, $z_c(\lambda w)=\lambda ^{-\gamma }z_c(w)$ with $\gamma =1+\nu +\xi$. Doing the same with the zooplankton growth rate (5.5) amounts to imposing the scaling

$$\begin{aligned} \int _0^{\infty }S(\lambda w,w')w'\big [p_c(w')+z_c(w')\big ]\,dw'= & {} \lambda ^{1-\xi }\int _0^{\infty }S(w,w')w'\big [p_c(w')\\&+\,z_c(w')\big ]\,dw', \end{aligned}$$

which, using the same argument as above, leads to $p_c(\lambda w)=\lambda ^{-\gamma }p_c(w)$.

An interesting by-product of this result is that the expressions for $M(w,w_*)$ and $G_z(w,w_*)$ have the same scaling form as those introduced in the analysis of phytoplankton in previous sections. Therefore we can obtain the steady state of the full system doing similar calculations. We will discuss this steady state first as obtained under the idealised division assumption and then as obtained for the general model.

### Steady state with idealised divisionprocess

We can again make the idealised division  assumption that cells divide exactly at size $w_*$ into two equal-size cells. As in the case of phytoplankton, this amounts to choosing $K_z(w,w_*,t)=G_z(w,w_*,t)\delta (w-w_*)$ and $Q(w|w')=\delta (w-w'/2)$, which transforms the population balance equation (2.10) into

5.11 $$\begin{aligned} \frac{\partial }{\partial t}z(w,w_*,t) = -\frac{\partial }{\partial w}\big [G_z(w,w_*,t)z(w,w_*,t)\big ] -M(w,w_*,t)z(w,w_*,t), \end{aligned}$$

valid in the interval $w_*/2\leqslant w\leqslant w_*$, with the boundary condition

5.12 $$\begin{aligned} 2G_z(w_*,w_*,t)z(w_*,w_*,t)=G_z(w_*/2,w_*,t)z(w_*/2,w_*,t). \end{aligned}$$

The expressions for the death and growth rates for zooplankton are formally the same as those for phytoplankton. Therefore the steady state size distributions of species abundances are given by

5.13 $$\begin{aligned} p(w,w_*)=p(w_*,w_*)\phi _{p}(w/w_*), \qquad z(w,w_*)=z(w_*,w_*)\phi _{z}(w/w_*), \end{aligned}$$

where

5.14 $$\begin{aligned} \phi _{p}(x)=\frac{a(N)-b}{a(N)x^{\alpha }-bx^{\beta }} \exp \left\{ \int _x^1\frac{m(y)}{a(N)y^{\alpha }-by^{\beta }}\,dy\right\} , \end{aligned}$$

$N$ being the steady state value of the nutrient concentration, and

5.15 $$\begin{aligned} \phi _{z}(x)=\frac{a_{pz}-b}{a_{pz}x^{1-\xi }-bx^{\beta }} \exp \left\{ \int _x^1\frac{m(y)}{a_{pz}y^{1-\xi }- by^{\beta }}\,dy\right\} \end{aligned}$$

with $a_{pz}$ given in Eq. (5.10).

The overall species abundances $p(w_*,w_*)$ and $z(w_*,w_*)$ can be obtained through Eqs. (5.3) and (5.6). For the phytoplankton, for instance, given that $p(w,w_*)=0$ for $w>w_*$,

$$\begin{aligned} p_c(w)=\int _w^{\infty }p(w_*,w_*)\phi _{p}(w/w_*)\,dx= w\int _0^1p\left( \frac{w}{x},\frac{w}{x}\right) \phi _{p}(x)\,\frac{dx}{x^2}. \end{aligned}$$

Now, given the scaling $p_c(\lambda w)=\lambda ^{-\gamma }p_c(w)$, this equation implies that $p(\lambda w_*,\lambda w_*)=\lambda ^{-\gamma -1} p(w_*,w_*)$, i.e.,

5.16 $$\begin{aligned} p(w_*,w_*)=\frac{p_0}{I_{p}(\gamma -1)}w_*^{-\gamma -1}, \end{aligned}$$

in terms of the functions

5.17 $$\begin{aligned} I_{p}(\eta )=\int _0^1x^{\eta }\phi _{p}(x)\,dx, \qquad I_{z}(\eta )=\int _0^1x^{\eta }\phi _{z}(x)\,dx. \end{aligned}$$

A similar argument yields

5.18 $$\begin{aligned} z(w_*,w_*)=\frac{z_0}{I_{z}(\gamma -1)}w_*^{-\gamma -1}. \end{aligned}$$

As in the case of phytoplankton alone, the level of nutrient at the steady state is determined by the boundary condition (3.13), which fixes the value of $a(N)$. There is a problem though. In this idealised version of a plankton community we are implicitly assuming an infinite biomass, because we are not imposing any lower nor upper limit on the size of cells. This translates into an infinite nutrient uptake by the phytoplankton,

$$\begin{aligned} \sigma (N,p)&=\frac{a(N)}{\theta }\int _0^{\infty }dw_*\,w_*^{1-\alpha -\xi } \int _0^{w_*}dw\,w^{\alpha }p(w,w_*) \\&=\frac{a(N)}{\theta }p_0\frac{I_{p}(\alpha )}{I_{p}(\gamma -1)} \int _0^{\infty }dw_*\,w_*^{1-\xi -\gamma }, \end{aligned}$$

which will then require an infinite amount of nutrient to survive.

In reality there will always be a minimum size $w_{\min }$ and a maximum size $w_{max}$, so if we introduce the factor

5.19 $$\begin{aligned} \varXi =\int _{w_{\min }}^{w_{\max }}dw_*\,w_*^{1-\xi -\gamma } \end{aligned}$$

and assume that all resource-related quantities diverge proportional to $\varXi$, we can rescale those quantities accordingly, so that they stay finite also in the limit of $w_{\min }\rightarrow 0$ and $w_{\max }\rightarrow \infty$. Hence we introduce a renormalised nutrient concentration $\hat{N}=\lim N/\varXi$, where the limit takes $w_{\min }\rightarrow 0$ and $w_{\max }\rightarrow \infty$, and similarly with other variables (a hat will henceforth denote these renormalised quantities). The dynamics of the nutrient (2.12), in terms of renormalised quantities, becomes^1^

5.20 $$\begin{aligned} \frac{d\hat{N}}{dt}=\hat{\varrho }(\hat{N})-\hat{\sigma }(\hat{N},p). \end{aligned}$$

Hence in the steady state the renormalised nutrient concentration satisfies $\hat{\varrho }(\hat{N})+\hat{\sigma }(\hat{N},p)$, which can be rewritten as

5.21 $$\begin{aligned} p_0=\frac{\theta \hat{\varrho }(\hat{N})I_{p}(\gamma -1)}{\hat{a}(\hat{N}) I_{p}(\alpha )}. \end{aligned}$$

Once we have determined $p_0$, the boundary condition

5.22 $$\begin{aligned} \int _{1/2}^1\frac{m(y)}{a_{pz}y^{1-\xi }-by^{\beta }}\,dy=\log 2 \end{aligned}$$

yields $a_{pz}$, which in turns determines $z_0$ via Eq. (5.10).

### Steady state with general division process

We can introduce a division rate for zooplankton $K_{z}(w,w_*)$ with similar properties as that for phytoplankton. The simplest choice is to take the same function—as it is conceivable that the dynamics of cell division does not depend on the feeding mechanism—or any other alternative, but in any case scaling (4.14) must hold for $K_{z}(w,w_*)$ as well. Also, we assume that the size distribution of daugher cells is described by the same function $Q(w|w')$.

Then we can introduce a similar splitting for zooplankton abundance

5.23 $$\begin{aligned} z(w,w_*,t)= {\left\{ \begin{array}{ll} z_l(w,w_*,t), &{}\quad w\ge w_+, \\ z_s(w,w_*,t), &{}\quad w\le w_+, \end{array}\right. } \end{aligned}$$

and write equations similar to (4.3) and (4.4). The steady state of those equations will be given by

5.24 $$\begin{aligned} p(w,w_*)=p(w_+,w_*)\psi _{p}(w/w_*), \qquad z(w,w_*)=z(w_+,w_*)\psi _{z}(w/w_*), \end{aligned}$$

where

5.25 $$\begin{aligned} \psi _{p}(x)&=\frac{a(N)\left( \frac{1+\delta }{2}\right) ^{\alpha }- b\left( \frac{1+\delta }{2}\right) ^{\beta }}{a(N)x^{\alpha }-bx^{\beta }} e_{p}(x)\varTheta _{p}(x), \end{aligned}$$

5.26 $$\begin{aligned} e_{p}(x)&=\exp \left\{ -\int _{\frac{1+\delta }{2}}^x\frac{k(y) +m(y)}{a(N)y^{\alpha }-by^{\beta }}\,dy\right\} , \end{aligned}$$

5.27 $$\begin{aligned} h_{p}(x)&= \int _{\frac{w_\text {th}}{w_*}}^1\frac{k(y)e_{p}(y)}{a(N)y^{\alpha }-by^{\beta }}q\left( \frac{x}{y}\right) \,dy, \end{aligned}$$

5.28 $$\begin{aligned} \varTheta _{p}(x)&= {\left\{ \begin{array}{ll} 1, &{}\quad x>\frac{1+\delta }{2}, \\ \displaystyle \int _0^x\frac{k(y)}{e_{p}(y)}\,dy, &{}\quad x<\frac{1+\delta }{2}, \end{array}\right. } \end{aligned}$$

and

5.29 $$\begin{aligned} \psi _{z}(x)&=\frac{a_{pz}\left( \frac{1+\delta }{2}\right) ^{1-\xi }- b\left( \frac{1+\delta }{2}\right) ^{\beta }}{a_{pz}x^{1-\xi }-bx^{\beta }} e_{z}(x)\varTheta _{z}(x), \end{aligned}$$

5.30 $$\begin{aligned} e_{z}(x)&=\exp \left\{ -\int _{\frac{1+\delta }{2}}^x\frac{k(y) +m(y)}{a_{pz}y^{1-\xi }-by^{\beta }}\,dy\right\} , \end{aligned}$$

5.31 $$\begin{aligned} h_{z}(x)&= \int _{\frac{w_\text {th}}{w_*}}^1\frac{k(y)e_{z}(y)}{a_{pz}y^{1-\xi }-by^{\beta }}q\left( \frac{x}{y}\right) \,dy, \end{aligned}$$

5.32 $$\begin{aligned} \varTheta _{z}(x)&= {\left\{ \begin{array}{ll} 1, &{}\quad x>\frac{1+\delta }{2}, \\ \displaystyle \int _0^x\frac{k(y)}{e_{z}(y)}\,dy, &{}\quad x<\frac{1+\delta }{2}. \end{array}\right. } \end{aligned}$$

Introducing the functions

5.33 $$\begin{aligned} J_{p}(\eta )=\int _0^1x^{\eta }\psi _{p}(x)\,dx, \qquad J_{z}(\eta )=\int _0^1x^{\eta }\psi _{z}(x)\,dx, \end{aligned}$$

and reproducing the arguments of Sect. 5.1, we obtain

5.34 $$\begin{aligned} p(w_+,w_*)=\frac{p_0}{J_{p}(\gamma -1)}w_*^{-\gamma -1}, \qquad z(w_+,w_*)=\frac{z_0}{J_{z}(\gamma -1)}w_*^{-\gamma -1}, \end{aligned}$$

with

5.35 $$\begin{aligned} p_0=\frac{\theta \hat{\varrho }(\hat{N})J_{p}(\gamma -1)}{\hat{a}(\hat{N}) J_{p}(\alpha )} \end{aligned}$$

and $z_0$ derived from (5.10), with $a_{pz}$ obtained through the boundary condition

5.36 $$\begin{aligned} \int _0^{\frac{1+\delta }{2}}\frac{h_{z}(y)}{e_{z}(y)}\, dy=1. \end{aligned}$$

What we can conclude from the analysis of the last two sections is that only two steady states are possible in this system in which zooplankton predate on phytoplankton: (a) a collapsed community in which at most a few species of phytoplankton—and possibly of zooplankton—survive; or (b) a community made of a continuum of species of sizes $0<w<\infty$ that align on a single power law spectrum, with an exponent $\gamma$ determined by the allometry of the phytoplankton growth rate and of the zooplankton predation rate.

It is interesting to realise how the different facts assemble together to yield this result. On the one hand, as zooplankton predation is the main cause of phytoplankton mortality, in order for several phytoplankton species to coexist the zooplankton community is forced to distribute their abundances on a power law. In turn, zooplankton grow by predation, and in order for several zooplankton species to coexist the phytoplankton community is forced to lie on the same power law. We see then that both communities sustain each other, and that biodiversity is both the cause and the consequence of the power law size spectrum.

## Scale invariance

In the last paragraph of Sect. 5 we gave an intuitive explanation of why both the phytoplankton and the zooplankton spectrum have to follow a power law in the steady-state. There is also a more formal explanation that we would like to exhibit in this section: the steady-state equations are scale-invariant in the sense that if an abundance spectrum $p(w,w_*)$, $z(w,w_*)$ is a solution of the steady-state equations for some level of nutrient $\hat{N}$, then so is the scale-transformed spectrum

6.1 $$\begin{aligned} p_\lambda (w,w_*)=\lambda ^{\gamma +1}p(\lambda w,\lambda w_*), \qquad z_\lambda (w,w_*)=\lambda ^{\gamma +1}z(\lambda w,\lambda w_*), \end{aligned}$$

for any positive $\lambda$. Thus solutions come in one-parameter families. The steady-state however is expected to be unique, and this implies that it must be scale invariant, which in turn implies that it must be of the power-law form

6.2 $$\begin{aligned} p(w,w_*)=w_*^{-\gamma -1}f_p(w/w_*) ~~\text { and }~~ z(w,w_*)=w_*^{-\gamma -1}f_z(w/w_*) \end{aligned}$$

for some scaling functions $f_p$ and $f_z$. These scaling functions were calculated explicitly in earlier sections and depend on some details of the model, but the power-law form of the abundances follows directly from the scale-invariance of the model and is insensitive to other details.

Note that the scaling discussed here, where rates and abundances can be expressed in terms of scaling functions of the ratio of individual size to characteristic size of the species, has also been employed by Giometto et al. (2013). It is different from the scaling discussed by Banavar et al. (2007) that scales size and area.

The viewpoint that the crucial property of the aquatic ecosystem is its scale invariance was previously taken by Capitán and Delius (2010), where a scale-invariant model for the fish part of the spectrum was presented. That paper did not model the dynamics of the plankton part of the spectrum but simply assumed that it was given by a power-law. The plankton model in this paper can be combined with the fish model of Capitán and Delius (2010) to give a dynamic scale-invariant model of the entire spectrum. What remains to be done is to explain why evolution, presented with the opportunity to fill a physical environment that itself exhibits scale invariance over many orders of magnitude, like an ocean or a large lake, would evolve organisms that preserve this scale invariance to a great degree.

## Discussion and conclusions

Traditionally, size-based models for the population dynamics of unicellular organisms concentrate either on cell-level processes like cell growth and cell division to describe the size distribution of cells within a species (Fredrickson et al. 1967; Diekmann et al. 1983; Heijmans 1984; Henson 2003; Friedlander and Brenner 2008), or they concentrate on population-level processes like predator–prey interactions to describe the abundance distribution across species of different characteristic size (Moloney and Field 1991; Gin et al. 1998; Armstrong 1999; Baird and Suthers 2007; Stock et al. 2008; Poulin and Franks 2010; Banas 2011; Ward et al. 2014). We have introduced a model that does both simultaneously: it resolves the distribution of cell sizes within a species and the distribution of biomass across species and thereby allows us to start from individual-level processes and their allometric scaling and from them derive population level phenomena like the power-law Sheldon spectrum. The only other works of a similar nature that we are aware of are Giometto et al. (2013) and Rossberg (2012).

At the cell level, our model combines a von Bertalanffy cell growth model with a flexible cell division model. This cell division model allows a sloppy size control, so that division can occur for a wide range of sizes. In addition, the two daughter cells do not necessarily have equal size but instead are described by a size distribution. Even though this is quite a general model for cell growth and cell division, we were able to give exact analytic solutions for the steady state cell size distribution. This is novel and may be useful also for studying size distributions of cells other than plankton cells. We also worked with an idealised version of the cell cycle (cells split into two identical daughter cells once they exactly double their size) in parallel to the more realistic model to show that the main conclusions of our paper do not depend on the details of the division model.

The most important aspect of our model is the coupling of the growth of a predator cell to the death of a smaller prey cell. This makes the cell growth rate depend on the abundance of prey and the cell death rate depend on the abundance of predators, leading to a non-linear model. It is remarkable that, in spite of this non-linearity, this coupling together of cells of all species allows an exact steady-state solution giving the size distributions for a continuum of coexisting species.

The model has the property that, at steady state, the coexistence of multiple unicellular plankton species and the Sheldon power-law size spectrum are two different manifestations of just one single phenomenon—‘two sides of the same coin’. This conclusion rests very much on the allometry of the four rates involved: the growth rates of phyto- and zooplankton, the death rate, and the predation kernel. The first and last of these allometries are supported by empirical data and have specified allometric exponents. However, the allometries of the zooplankton growth and death rates have to emerge from the predator–prey interactions at steady state, and are technical outcomes of our modelling. In summary, one can assume in the model any one of the following properties: (a) allometry of the rates, (b) coexistence of multiple species, and (c) a power-law community size spectrum. Then, from this, the other two properties can be derived.

While we have been able to show analytically in this paper that the model predicts a coexistence steady state that agrees with the Sheldon spectrum, we have not discussed the stability of this steady state against small perturbations. Due to the complicated form of the steady state solution, an analytic stability analysis is not feasible. We have therefore performed the numerical calculations and have reported on them in a less analytically demanding paper (Law et al. 2016). The numerical results show that additional stabilising terms, like for example an extra density dependence of the predation rate, need to be added to the model to stabilise the steady state. This is in agreement with observations in Maury and Poggiale (2013, section 4.2). As those stabilising terms are reduced, the system undergoes a Hopf bifurcation during which the steady-state becomes unstable and the new attractor is an oscillatory state describing waves of biomass moving up the size spectrum. When averaged over time, these oscillations average out to a power-law abundance.

Our analytical model presented in this paper is trait-based rather than species-based, which means that, rather than taking a finite set of species, it uses a continuum of species distinguished by a continuous trait variable, in our case the maximum size of a cell of that species. All analytical results in this paper very much rely on the existence of this continuum of species, which is clearly an idealisation of a real aquatic ecosystem that can only contain a finite set of species. In that case the community abundance can never be an exact power law, and therefore also the resulting allometric scaling can not be exact. One may wonder whether the qualitative results of this paper will continue to apply.

To test that, we have carried out numerical simulations of a version of the model (Law et al. 2016) with a finite number of species. A single zooplankton species feeding on an assemblage of twenty phytoplankton species drives its phytoplankton prey to very low densities while on the path to extinction itself, leaving a community far from being described by a Sheldon spectrum. However, increasing the number of zooplankton species to nine, distributed over a range of characteristic cell sizes, leads to a community closer to a Sheldon spectrum. This is because with more zooplankton species there is a closer approximation to the scaling needed for predation mortality in the prey, in conjunction with the scaling needed for growth in the predator. Moreover, the steady state is locally asymptotically stable. This is quite different from the results on random matrices (May 1972; Allesina and Tang 2015) which have been interpreted in ecology as making it less likely for a community to be stable as species-richness and connectance increase. Ecologists have looked extensively for more realistic network structures that could counter such instability in complex communities, e.g. Neutel et al. (2002), Neutel et al. (2007), Neutel and Thorne (2014), James et al. (2015) and Jacquet et al. (2016). Our results in Law et al. (2016) suggest that stable, unicellular, ecological networks with Sheldon-like structure cannot be achieved unless enough species are present to generate approximations to the scalings in predation.

In previous models investigating the continuous coexistence of species, e.g., Gyllenberg and Meszéna (2005) and Sasaki (1997), it had been found that even when a model has a coexistence steady state that is dynamically stable, this steady state is unstable against any small structural change to the model, and regions of exclusion develop in trait space in which no species can exist. However, the results in Law et al. (2016) show that our model does not suffer from this structural instability. When we go from the exact power-law death and growth rates of the continuous model to the approximate power-law in the presence of a finite number of species, the steady-state remains stable and the abundance of these species still approximately follows the Sheldon spectrum power law. How much of this structural stability is due to the extra density dependence in the predation rate and how much of it is due to the fact that we explicitly model the growth of individuals whereas previous works assumed that all individuals of a species had the same size, remains to be investigated.

Our model works with a single trait variable. It ignores all other characteristics that distinguish different species, except whether it is an autotroph or a heterotroph. Clearly the model could be made more realistic by also distinguishing between different functional types, for example between diatoms and dinoflagelates. Also, it would be easy to include mixotrophs without changing the conclusions of our model. However we wanted our model to be the simplest conceptual model that clarifies how coexistence on a Sheldon spectrum emerges. For the same reason, we included only a single resource described by a very simple equation, whereas in reality the resource dynamics are complicated and very seasonal.

One question that we have not addressed in this paper is the reason for the observed allometric scaling of the phytoplankton growth rate and the predation rate. As these are at the basis of our derivation of coexistence and the Sheldon spectrum, finding an explanation for them would be very interesting. The allometry of the phytoplankton growth rate may possibly be imposed by physical constraints. Allometric scaling has been studied a lot over the last 30 years, starting with the classics (Calder 1984; Bonner and McMahon 1983; Schmidt-Nielsen 1984) and with important contributions by West et al. (1997), Banavar et al. (1999), Banavar et al. (2014), Kooijman (2010) and many others.

The predation kernel, on the other hand, combines two ingredients: a preferred prey size and a foraging term. Although there may also be physical constraints for the latter, both ingredients are to a great extend behavioural—hence subject to evolution. Take the preference for a prey size, for instance. It is hard to believe that if the abundance of the preferred prey is seriously depleted the predator will not adapt its consumption habits to keep a sufficient food supply. We believe that, instead of an input, the predation kernel should be an emergent feature, consequence of an underlying evolutionary principle that guides efficient predation habits. We have to leave this interesting question for the future.

## Appendix: Check of scale-invariance of steady-state equations

In this appendix we will verify our claim that the pair of functions

7.1 $$\begin{aligned} p_\lambda (w,w_*)=\lambda ^{\gamma +1}p(\lambda w,\lambda w_*),\qquad z_\lambda (w,w_*)=\lambda ^{\gamma +1}z(\lambda w,\lambda w_*), \end{aligned}$$

where

7.2 $$\begin{aligned} \gamma =1+\nu +\xi , \end{aligned}$$

solve steady-state equations provided the original functions $p(w,w_*)$, $z(w,w_*)$ do for the same nutrient  value $\hat{N}$.

The steady state equations, obtained by setting the time derivative to zero in the dynamical equations (2.10) for $p(w,w_*)$, (5.4) for $z(w,w_*)$, and (5.20) for $\hat{N}$ are

7.3 $$\begin{aligned} \frac{\partial }{\partial w}\big [G_p(w,w_*)p(w,w_*)\big ] =&2\int _0^{w_*}Q(w|w')K(w',w_*)p(w',w_*)\,dw' \nonumber \\&-K(w,w_*)p(w,w_*)-M(w,w_*)p(w,w_*). \end{aligned}$$

7.4 $$\begin{aligned} \frac{\partial }{\partial w}\big [G_z(w,w_*)z(w,w_*)\big ] =&2\int _0^{\infty }Q(w|w')K_z(w',w_*)z(w',w_*)\,dw' \nonumber \\&-K_z(w,w_*)z(w,w_*)-M(w,w_*)z(w,w_*), \end{aligned}$$

7.5 $$\begin{aligned} \hat{\varrho }(\hat{N})=&\hat{\sigma }(\hat{N},p). \end{aligned}$$

To check that $p_\lambda (w,w_*)$, $z_\lambda (w,w_*)$ solve these equations we simply substitute them. Let us start with (7.3) and consider each term individually. The left-hand side gives

7.6 $$\begin{aligned} \begin{aligned} \frac{\partial }{\partial w}\left[ G_p(w,w_*)p_\lambda (w,w_*)\right]&=\frac{\partial }{\partial w}\left[ G_p(w,w_*)\lambda ^{\gamma +1} p(\lambda w,\lambda w_*)\right] \\&=\lambda \frac{\partial }{\partial (\lambda w)} \left[ \lambda ^{\xi -1}G_p(\lambda w,\lambda w_*)\lambda ^{\gamma +1} p(\lambda w,\lambda w_*)\right] \\&=\lambda ^{\gamma +1+\xi }\frac{\partial }{\partial (\lambda w)} \left[ G_p(\lambda w,\lambda w_*)p(\lambda w,\lambda w_*)\right] , \end{aligned} \end{aligned}$$

where we used the scaling property (2.6) of the growth rate $G_p(w,w_*)$. The first term on the right-hand side of (7.3) gives

7.7 $$\begin{aligned} \begin{aligned} 2\int _0^{w_*}&Q(w|w')K(w',w_*)p_\lambda (w',w_*)\,dw'\\&=2\int _0^{\lambda w_*}\lambda Q(\lambda w|\lambda w')\lambda ^\xi K(\lambda w',\lambda w_*)\lambda ^{\gamma +1}p(\lambda w',\lambda w_*) \,\lambda ^{-1}d(\lambda w')\\&=\lambda ^{\gamma +1+\xi } 2\int _0^{\lambda w_*}Q(\lambda w|\lambda w') K(\lambda w',\lambda w_*)p(\lambda w',\lambda w_*) \,d(\lambda w'), \end{aligned} \end{aligned}$$

where we used the scaling properties (2.8) and (3.5). The second term gives

7.8 $$\begin{aligned} \begin{aligned} -K(w,w_*)p_\lambda (w,w_*)&=-\lambda ^\xi K(\lambda w,\lambda w_*)\lambda ^{\gamma +1} p(\lambda w,\lambda w_*)\\&=-\lambda ^{\gamma +1+\xi } K(\lambda w,\lambda w_*)p(\lambda w,\lambda w_*) \end{aligned} \end{aligned}$$

again due to the scaling property (3.5) of the division rate. Finally, using the expression (5.2) for the mortality rate, the last term gives

7.9 $$\begin{aligned} \begin{aligned} -M(w,w_*)p_\lambda (w,w_*)&=-\left[ \int _0^\infty S(w',w)\int _0^\infty z_\lambda (w',w_*')\,dw_*'\,dw'\right. \\&\quad \left. + w_*^{-\xi }m_b(w/w_*)\right] p_\lambda (w,w_*)\\&=-\Big [\int _0^\infty \lambda ^{-\nu }S(\lambda w',\lambda w)\\&\qquad \int _0^\infty \lambda ^{\gamma +1}z(\lambda w',\lambda w_*')\, \lambda ^{-1}d(\lambda w_*')\,\lambda ^{-1}d(\lambda w')\\&\quad + \lambda ^\xi (\lambda w_*)^{-\xi }m_b(\lambda w/\lambda w_*)\Big ] \lambda ^{\gamma +1}p(\lambda w,\lambda w_*)\\&=-\lambda ^{\gamma +1+\xi }M(\lambda w,\lambda w_*) p_\lambda (\lambda w,\lambda w_*). \end{aligned} \end{aligned}$$

We substituted both $p_\lambda$ and $z_\lambda$, used the scaling property of the predation rate $S(w,w')$ that follows from Eq. (5.1) and the relation (7.2) between the exponents.

Putting these four terms back together, we see that the resulting equation is the same as the original equation evaluated at scaled weights $\lambda w$ and $\lambda w_*$, up to an overall factor of $\lambda ^{\gamma +1+\xi }$. Given that the original equation holds for all weights *w* and $w_*$, this shows that the transformed equation is equivalent to the original one, establishing its scale invariance.

In the Eq. (7.4) for the zooplankton abundance the left-hand side involves the zooplankton growth rate given in Eq. (5.5) and we first determine its behaviour when $p_{\lambda }$ and $z_{\lambda }$ replace the original functions:

7.10 $$\begin{aligned} \begin{aligned} G_z(w,w_*)&=\int _0^\infty S(w,w')\varepsilon w' \left[ \int _0^\infty (p_\lambda (w',w_*')+z_\lambda (w',w_*')) dw_*'\right] dw'\\&\quad -bw_*^{1-\xi }\left( \frac{w}{w_*}\right) ^{\beta }\\&=\int _0^\infty \lambda ^{-\nu }S(\lambda w,\lambda w')\varepsilon \lambda ^{-1}(\lambda w') \left[ \int _0^\infty \lambda ^{\gamma +1}(p(\lambda w',\lambda w_*')\right. \\&\quad \left. +z(\lambda w',\lambda w_*')) \lambda ^{-1}d(\lambda w_*')\right] \lambda ^{-1}d(\lambda w')\\&\quad -b\lambda ^{-1+\xi }(\lambda w_*)^{1-\xi } \left( \frac{\lambda w}{\lambda w_*}\right) ^{\beta }\\&=\lambda ^{\xi -1}G_z(\lambda w,\lambda w_*). \end{aligned} \end{aligned}$$

Thus under the scale transformation the left-hand side of the zooplankton equation (7.4) becomes

7.11 $$\begin{aligned} \begin{aligned}&\frac{\partial }{\partial w}\left[ \lambda ^{\xi -1}G_z(\lambda w,\lambda w_*) \lambda ^{\gamma +1}z(\lambda w,\lambda w_*)\right] \\&\qquad =\lambda ^{\gamma +1+\xi }\frac{\partial }{\partial (\lambda w)} \left[ G_z(\lambda w,\lambda w_*)z(\lambda w,\lambda w_*)\right] . \end{aligned} \end{aligned}$$

The terms on the right-hand side transform just like those in the phytoplantkon equation. So again we find that the transformed equation is the same as the original equation at rescaled weights up to an overall factor of $\lambda ^{\gamma +1+\xi }$.

Finally, in the renormalised resource equation (7.5) the left-hand side does not depend on weights or plankton abundances, so is invariant under the scale transformation. The right hand side transforms to

7.12 $$\begin{aligned} \begin{aligned} \hat{\sigma }(\hat{N},p_\lambda )&=\lim \dfrac{\frac{\hat{a}(\hat{N})}{\theta } \int _{w_{min}}^{w_{max}}w_*^{1-\alpha -\xi }\int _0^{w_*} w^\alpha p_\lambda (w,w_*)dw dw_*}{\int _{w_{min}}^{w_{max}}w_*^{1-\xi -\gamma }dw_*}\\&=\lim \dfrac{\frac{\hat{a}(\hat{N})}{\theta } \int _{\lambda w_{min}}^{\lambda w_{max}}\lambda ^{-1+\alpha +\xi } (\lambda w_*)^{1-\alpha -\xi }\int _0^{\lambda w_*} \lambda ^{-\alpha }(\lambda w)^\alpha \lambda ^{\gamma +1}p(\lambda w,\lambda w_*) \lambda ^{-1}d(\lambda w)\lambda ^{-1} d(\lambda w_*)}{\int _{\lambda w_{min}}^{\lambda w_{max}}\lambda ^{-1+\xi +\gamma } (\lambda w_*)^{1-\xi -\gamma }\lambda ^{-1}d(\lambda w_*)}\\&=\hat{\sigma }(\hat{N},p) \end{aligned} \end{aligned}$$

and thus is also invariant, meaning the entire equation is invariant. This completes the proof that all the steady-state equations are scale-invariant.

## Footnotes

1 In these expressions $\hat{a}(\hat{N})=a_{\infty }\hat{N}/(\hat{r}+\hat{N})$ and $\hat{\varrho }(\hat{N})=\hat{\varrho }_0(1-\hat{N}/\hat{N}_0)$.
